# Evaluation of the Technical Performance of Football Players in the UEFA Champions League

**DOI:** 10.3390/ijerph17020604

**Published:** 2020-01-17

**Authors:** Qing Yi, Miguel-Ángel Gómez-Ruano, Hongyou Liu, Shaoliang Zhang, Binghong Gao, Fabian Wunderlich, Daniel Memmert

**Affiliations:** 1 School of Physical Education and Sport Training, Shanghai University of Sport, Shanghai 200438, China; 2 Shanghai Key Lab of Human Performance, Shanghai University of Sport, Shanghai 200438, China; 3 Facultad de Ciencias de la Actividad Física y del Deporte (INEF), Universidad Politécnica de Madrid, 28040 Madrid, Spain; 4 Institute of Training and Computer Science in Sport, German Sport University Cologne, 50933 Cologne, Germany; 5 School of Physical Education & Sports Science, South China Normal University, Guangzhou 510631, China; 6 Division of Sport Science & Physical Education, Tsinghua University, Beijing 100084, China

**Keywords:** technical performance profile, situational variable, playing position, football, soccer, match analysis

## Abstract

This study aimed to assess the technical match performance of top-class football players in a long-term perspective. Technical performance profiles of players according to five playing positions (central defender, full back, wide midfielder, central midfielder, forward) and five situational variables (competition stage, match location, quality of team, quality of opponent, match outcome) were established. Technical match data of players in the UEFA Champions League from season 2009–2010 to 2016–2017 were analyzed. The true effects of positional and situational variables on players’ technical performance were evaluated by the non-clinical magnitude-based inference. Results showed that the effect of *competition stage* on player’s performance was negligible. *Quality of team*, *quality of opponent* and *match outcome* revealed the strongest effects on player’s performance (ES: −0.42 ± 0.10–0.59 ± 0.10) while the effect of *match location* was relatively lower (ES: −0.32 ± 0.10–0.23 ± 0.07). The number of variables that showed statistical differences under five competing contexts for wide midfielders and forwards were higher than those of central defenders, full backs, and central midfielders. Differences of players’ match performance could mainly be identified in variables related to goal scoring, passing, and organizing, these findings may provide important insights for coaches and analysts during the match preparation and training session.

## 1. Introduction

The complexity of football match performance can be reduced by using performance analysis techniques, presenting the results in systematic ways, and systematically integrating them into the coaching process [1,2]. This can be considered as valuable feedback for coaches, players, and sport researchers [3,4].

Whether performance analysis can be good feedback or an educational tool depends on the type and quality of the methods used [5]. An accurate and reliable performance profile may improve the efficiency of the analysis procedure [6,7], and can provide useful feedback that can be easily understood by sports practitioners [8]. Performance profiling is a descriptive analysis that brings a collection of valid and reliable psychological, physical, and technical indicators together to characterize the overall performance of players and teams [9,10]. However, the properties of match performance indicators may vary along the matches played as situational variables have an influence on them [11,12]. Therefore, the data from a single match cannot represent a player’s or a team’s typical performance [9,13]. The individual match effects may generate additional variance in the data of a single match and fail to produce a significant difference in the comparison between groups [14]. Accordingly, Hughes et al. [6] stated that the nature of the data and the performers are the two main factors that should be taken into account when considering the number of matches required. The typical performance of a player/team may only be represented by the data from a large number of matches using the technique of performance profiling. Due to the availability of data, the profiling subject has developed over time from the analysis of single players or teams to more comprehensive analysis including a larger number of players or teams [15]. Previously, median and 95% confidence intervals [9,16] were used to present the typical performance of subjects and its spread of performance, while currently profiling techniques are commonly based on mean and 95% confidence intervals [17,18].

Due to the nature of complexity and highly dynamic behaviour in football matches [18,19,20,21], the match performance of players/teams is not only influenced by technical, tactical, mental [22,23], and physiological factors [24], but also by different situational factors [25,26]. Match performance of players/teams cannot be generalized in all contexts [15] and introducing the situational variables into performance profile can make it more comprehensive and systematic [17]. Match location, team quality, quality of opposition, and match outcome are examples of situational variables that have been investigated so far [17,18,27]. The quality of the opponent is considered the main factor for variation in match performance [28]. However, given the high dynamic nature of the match play highlighted previously, there is also an obvious opening for further research to take the interactions between playing positions and competing situations into deeper consideration. In the current study, sufficient match observations make it possible to take player positions into account to establish performance profiles according to different playing positions under different competing situations [17,27,29]. As players from different playing positions have different tasks in a football game, the general evaluation of all players’ match performance will result in a loss of a lot of valuable information. Therefore, a more useful way is a detailed analysis of the performance of players with proper regard to the particularity of different playing positions and the effect of situational variables.

In contrast to prior studies using data from domestic leagues [17,30,31], the evaluation of data from the UEFA Champions League makes it possible to evaluate differences of performance in different competition stages (*group* vs. *knockout stage*). Moreover, the database allows for comparisons between performances in international matches compared to domestic matches investigated in earlier studies. In order to add these aspects to the current research on performance analysis in football, the current study aimed to establish technical performance profiles of players in the UEFA Champions League from season 2009–2010 to 2016–2017 by comparing the between-player differences in match performance in a long-term perspective (8 full seasons) according to players’ specific field positions and incorporating five situational variables: *competition stage* (*group stage/knockout stage*), *match location*, *quality of team*, *quality of opposition* and *match outcome*.

## 2. Materials and Methods

### 2.1. Data and Reliability

Technical performance-related match data of players in the UEFA Champions League from season 2009–2010 to 2016–2017 (1000 matches, 768 matches from group stage, 232 matches from knockout stage) were collected for analysis. Match data were obtained from a public-accessed football statistics website “whoscored.com” (https://www.whoscored.com). Data resource of the website is the sports analytics company OPTA Sports (London, UK). The reliability of OPTA in coding players’ match actions and events has already been successfully tested as highly reliable [32]. Because of the specificity of position for goalkeepers and a very limited applicability of technical variables to the performance of goalkeepers, match data for this position was excluded from the sample. Moreover, only the players being part of the starting line-up were included, which finally limited the database to 5136 players (3693 players from group stage, 1443 players from knockout stage) and generated 14,437 full match observations (11,095 from group stage, 3342 from knockout stage). All players in the sample were divided into 5 groups according to their playing positions [17,33]: central defender (group stage: *N*_1_ = 1012 players, *n*_1_ = 2811 full match observations, knockout stage: *N*_2_ = 380 players, *n*_2_ = 821 observations), full back (*N*_1_ = 939, *n*_1_ = 2657, *N*_2_ = 337, *n*_2_ = 772), wide midfielder (*N*_1_ = 711, *n*_1_ = 1214, *N*_2_ = 216, *n*_2_ = 351), central midfielder (*N*_1_ = 1326, *n*_1_ = 3081, *N*_2_ = 511, *n*_2_ = 963), and forward (*N*_1_ = 734, *n*_1_ = 1332, *N*_2_ = 237, *n*_2_ = 435). The current study was conducted in accordance with the Declaration of Helsinki and approved officially by the ethics committee of the Shanghai University of Sport (11DZ2261100).

### 2.2. Technical Variables and Situational Variables

Twenty-five technical performance-related match actions or events were chosen as variables in the present study and were divided into four groups (Table 1) based on previous studies [18,34,35,36]. Definitions of these variables can be found in the previous studies [11,20,36]. The technical match data was analyzed under the following five situational variables: (1) *competition stage*: *group stage and knockout stage*; (2) *match location: home and away*; (3) *quality of team*: teams that *qualified* into the knockout stage and teams that *didn’t qualify* into the knockout stage; (4*) quality of opponent*: *opponents that qualified* into the knockout stage and *opponents that didn’t qualify* into the knockout stage; and (5) *match outcome*: *win*, *draw* and *lose*. Due to the limitation of the sample size from knockout stage, except for the situational variable of competition stage, only the match data of players in the group stage were included in the analysis of the other four situational variables.

### 2.3. Statistical Analysis

According to the central limit theorem, the sampling distribution of the match performance statistics based on the large database will be normal, and the variance will be homogeneous as well. Thus, the test for data normality distribution and homogeneity of variance was not performed in the process of statistical analysis [37]. After the screening of missing values and outliers, count values of 25 technical performance-related actions or events of players were transformed into standardized scores (Z-Score) and were unified into the same scale by the formula T = 10Z + 50 [27,38]. Match performance of players from five positions were compared by means of adjusted values respectively accounting to five situational variables and were plotted into radar charts. The standardized scores (Z-Score) were then transformed and unified using the statistical software IBM SPSS Statistics 22 for Windows (IBM Corp., Armonk, NY, USA) and plotted into radar charts using the Microsoft Excel 2007 program (Microsoft, Redmond, WA, USA). The non-clinical magnitude-based inference (MBI) was used to identify the differences of match performance of players. Differences were evaluated by using the standardized smallest worthwhile change which was calculated by 0.2 times the between-subject standard deviation [39]. Comparisons between groups were conducted using the spreadsheet developed by Hopkins and 90% confidence intervals were used to make the inferences [40,41]. Magnitude of clear differences was considered as follows: trivial, 0–0.2; small, 0.2–0.6; moderate, 0.6–1.2; large, 1.2–2.0; and very large, >2.0 [39,42]. The possibility of the effect to be clear was defined as follows: 25–75%, possibly; 75–95%, likely; 95–99.5%, very likely; and 99.5–100%, most likely [42].

## 3. Results

**Figure 1:**
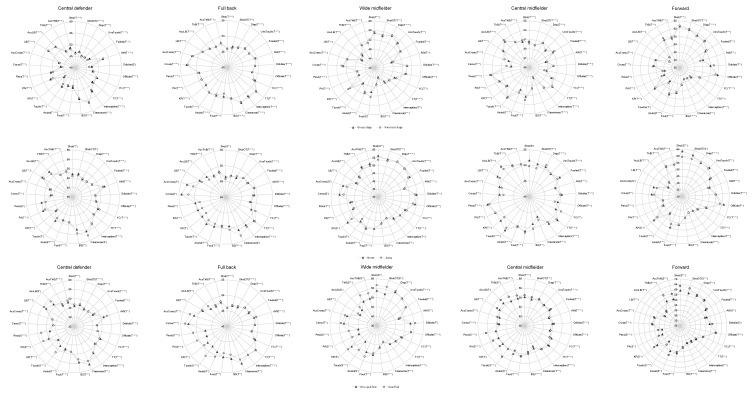

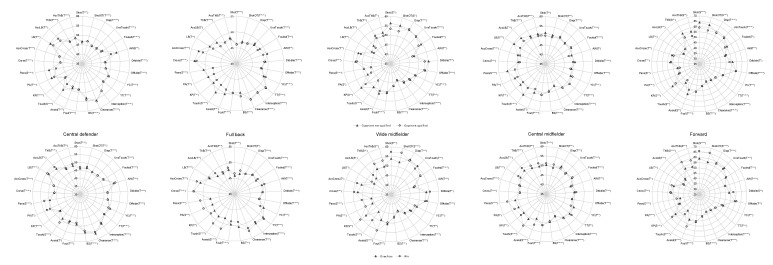
Comparison of the performance profiles of different position’s players under five situational variables. Notes: letters in parentheses denote the magnitude: t = trivial; s = small. Asterisks indicate the likelihood for the magnitude of the true difference in means as follows: * possible; ** likely; *** very likely; **** most likely. Abbreviations: AccCross = accurate cross pass; AccLB = accurate long ball; AccThB = accurate through ball; AW = aerial won; BS = blocked shot; Disp = player is dispossessed on the ball by an opponent-no dribble involved; KP = key pass; LB = long ball; PA = pass accuracy in %; ShotOT = shot on target; ThB = through ball; TT = total tackle; UnsTouch = Unsuccessful touch; YC = yellow card.

Comparisons in technical variables between players from five positions according to five situational variables are presented in Figure 1 and Table 2. Figure 1 shows a graphical representation of the performance profiles including the results of magnitude-based inferences. Table 2 gives a summary of all technical variables that revealed non-trivial differences. The full set of descriptive statistics of match performance profiles can be found in the Supplementary Materials file.

There is a new finding concerning the influence of competition stage on player performance that did not appear in the previous research due to the investigation of domestic matches [17,30] or the lack of knockout matches in international tournaments [43,44]. In all variables from five positions between players from *group stage* and players from *knockout stage* no clear differences were identified, except for fouling. The comparisons regarding the other four situational variables in the *group stage* revealed more substantial differences in performance. The number of variables showing non-trivial differences between *home* games and *away* games were limited across all playing positions. Especially in the case of central midfielders, only *shots* showed a non-trivial difference. Central defenders and full backs made more *clearances* when playing *away* than when playing at *home* in the *group stage*. Their performance in variables related to passing and organizing (*passes* and *crosses*) in *home* games was better than in *away* games. The differences of match performance of wide midfielders and forwards between *home* and *away* games were mainly focused on variables related to goal scoring and variables related to passing and organizing, while the central midfielders only showed clear difference in *shots*. A similar trend was found by comparing player’s match performance when considering the effect of *quality of team*, *quality of opponent* and *match outcome*. Across all positions from qualified teams, players playing against non-qualified teams and players of a winning team showed better performances in variables related to passing and organizing than their counterparts (players from non-qualified teams, players playing against qualified teams, and players playing in games lost/draw). Wide midfielders and forwards showed clear differences in variables related to goal scoring (*shots* and *shots on target*) in these three competing contexts. In addition, forwards from qualified teams and from winning teams made more dribbles when compared with their counterparts from non-qualified teams and playing in *games lost/draw*. Central defenders obtained more *aerials won* when playing with non-qualified teams than when playing with qualified teams.

Another important result that can be found in Table 2 is that *touches* and *passes* are the only two variables that showed clear differences for players of all positions when taking *quality of team*, *quality of opponent,* and *match outcome* into account. *Touches* and *passes* of wide midfielders showed bigger differences in these three competing situations compared with those from the other four playing positions. Moreover, match performance of players from all five playing positions in *touches* and *passes* under the situational variable of team quality showed greater differences than those under the other two situational variables. In the present study, no clear differences were detected for the match variables *yellow cards*, *dispossessed*, *unsuccessful touches*, *total tackles*, *interceptions*, *blocked shots*, *fouled,* and *offsides*, neither across playing positions, nor under all five situational variables.

## 4. Discussion

The current study established technical performance profiles of players from the UEFA Champions League based on a large sample (*N* = 1000 matches from 8 seasons) to identify the differences of technical performance between players across different situational variables and playing positions. Thus, the interaction between situational and positional variables were investigated.

Generally, all variables showed small or trivial differences across five situational variables and five playing positions. The effects of situational variables on the technical performance of players from different playing positions were lower than in previous studies [31,45]. This finding may be due to the fact that the analysis of long-term data may reduce the impact of situational variables on players’ performance due to the higher stability of the performances during eight seasons under the different contexts. The differences of player’s technical performance could mainly be identified in variables related to passing and organizing, especially *touches* and *passes*. In particular, passing and organizing abilities are time-space and task-related variables. The different profiles may reflect that these are key determinants of performance in elite football during the last few years [45]. Therefore, the present study helps to reveal the interactions between playing positions and situational variables in these two technical variables.

To the best of our knowledge, there is no research that has examined the differences of player’s performance between group stage and knockout stage of the UEFA Champions League so far. Our database provided us with the possibility to analyze the performance of players in matches from different competition stages. Clear differences when comparing matches from group and knockout stages would be expected given the special characteristics of knockout matches such as the high importance and the presence of higher quality teams than the group stage. Surprisingly, we found that there was no statistical difference in player’s performance between *group stage* and *knockout stage*, except for *fouls* in the group of wide midfielders. This demonstrates a high consistence of player’s performance between *group stage* and *knockout stage*, although these two competition stages are characterized by different characteristics of matches. Wide midfielders might have more defensive tasks when playing against strong opponents compared to when playing against weak opponents as they face higher defensive pressure [46], which could explain why wide midfielders from knockout stage committed more fouls than wide midfielders from group stage.

Recent research identified that *match location* had a significant influence on technical variables [17], although it had limited impact on physical variables [47]. The effect of *match location* on players’ performance in our database is lower than those of reported in prior studies. Central defenders and full backs made more *clearances* and less *passes* and *crosses* in *away* games than in *home* games, which may reveal that home teams tend to employ a more aggressive strategy. In home games, central defenders are involved in the organizing and attacking process, and full backs are moving forward frequently into the attacking third to make *crosses* for teammates. In contrast, away teams will face more defensive pressure, thus defenders have to make more *clearances* to block opponent’s attack. The fact that wide midfielders, central midfielders and forwards from home teams obtained more shots opportunities than their counterparts from away teams supports the theory that home teams play a more aggressive and offensive strategy. Surprisingly, central midfielders did not show clear differences in variables related to passing and organizing between *home* games and *away* games, which may indicate that the performance of central midfielders in passing and organizing related variables is stable regardless of match location.

Three of the situational variables (*quality of team*, *quality of opponent,* and *match outcome*)—although covering different aspects—are in some way connected to teams’ strength. Consequently, similar trends in player’s technical performance were found in these three contexts. Moreover, they had a relatively greater influence on players’ technical performance than competition stage and match location. Technical performance of players from all playing positions in these three competing contexts showed clear differences in variables related to passing and organizing. Similar findings were reported in a previous study on Spanish La Liga [17], which shows that players’ performance was consistent between a domestic league and international competition. The important role of variables related to passing and organizing can be explained as follows: Stronger teams have a higher ability to retain ball possession, to control the game, and have a higher initiative to score goals instead of preventing goals. Our findings revealed that either strong teams are more likely to adopt a possession-based playing style, or that improving players’ ability in the aspect of passing and organizing can help to achieve a better match performance for football teams in the *group stage*. Moreover, forwards’ performance in *shots*, *shots on target,* and *dribbles* showed clear differences within these three contexts, which might be a result of the differences of skill level between forwards and the support they got from midfielders based on the advantage of possession. However, wide midfielders from qualified teams, playing against non-qualified teams and playing in winning games gained more *shots* and *shots on target* than wide midfielders from non-qualified teams, playing against qualified teams and playing in *draw/losing* games. This indicates that wide midfielders from stronger teams had more opportunities to participate in the offensive phase and to invade from wide to inside, hence they are likely to get more scoring chances [17].

We also found that *touches* and *passes* were the only two variables in which match performance of players from all playing positions showed clear differences when considering the effect of *quality of team*, *quality of opponent,* and *match outcome.* This provides us with the opportunity to explore the interaction between five playing positions and three situational variables. Wide midfielders showed the biggest differences in *touches* and *passes* between stronger teams and weaker teams. A possible reason is that wide midfielders from stronger teams played an important role in the organizing and attacking phase, while wide midfielders from weaker teams relatively need to take on more defensive tasks. The magnitude of differences in *touches* and *passes* for players from all five playing positions seems to be mostly influenced by the effect of *quality of team* within these three situational variables. Most variables related to attacking and defending showed unclear differences across different playing positions and different situational variables, which indicates that the differences of players’ match performance does not mainly result from attacking and defending abilities, but from the ability of managing the game, keeping hold of the ball, and creating scoring opportunities [33].

## 5. Conclusions

The present study contributes to the current research on performance analysis in top-class football [48] by establishing more comprehensive and detailed technical profiles to examine the interaction of positional and situational variables on players’ technical performance. This could be an important step to provide information on players’ match performance and their interactions. Generally, the magnitudes of differences for all variables were displayed at a low level (small or trivial) based on a large dataset. Match location, quality of team, quality of opponent, and match outcome demonstrated significant effect on players’ technical performance while the effect of competition stage on players’ technical performance was strongly limited. Strength-related situational variables showed similar trends and had a relatively greater influence on players’ technical performance than match location. The technical performance of each playing position also varies under different competing contexts. Wide midfielders showed the biggest differences in variables related to passing and organizing within strength-related situational variables and the *quality of team* had a bigger impact on players’ match performance in variables related to passing and organizing compared to *quality of opponent* and *match outcome*. The differences of players’ technical performance could mainly be identified in variables related to goal scoring and variables related to passing and organizing, while there were no clear differences in most attacking and defending related variables. Technical performance of individual players can be evaluated by integrating their match data into the performance profiles, which may provide a valuable tool for player recruitment and talent identification. Moreover, these technical performance profiles can also be used during pre-match preparation, while considering the conditions of the next match, and during post-match assessment to develop position-specific interventions in the coaching process.

However, there are still opportunities to expand the level of this research by adding relevant information in future research. There were 625 pairwise comparisons conducted to identify the differences in technical performance of players from five positions under five competing situations, which may probably result in an increase of type I errors. Thus, this issue should be addressed in further research. Previous studies have identified the influence of match status (e.g., score and time left) on the players’ match performance. But as all data used within this study is aggregated data reflecting the whole match time, the match status hasn’t been considered as an additional situational variable in this study. Another aspect that could be valuable for future research on performance profiles of football players is positional data. A growing availability of positional data has led to innovations in match analysis in recent years. The investigation of advanced key performance indicators based on positional data can help to gain additional insights to performance of football players. Future research on performance profiles might profit from this work by including variables based on positional data into the performance profiles and thus expanding performance analysis from technical variables to tactical variables.

## Figures and Tables

**Table 1:** The classification of technical variables.

Categories	Variables
**Goal scoring**	*Shot, Shot on target.*
**Attacking**	*Dispossessed, Unsuccessful touch, Fouled, Aerial won, Dribble, Offside.*
**Defending**	*Yellow card, Total tackle, Interception, Clearance, Blocked shot, Foul.*
**Passing and organizing**	*Assist, Touch, Key pass, Pass accuracy (%), Pass, Cross, Accurate cross, Long ball, Accurate long ball, Through ball, Accurate through ball.*

**Table 2:** Statistical differences of players’ match performance across five playing positions and five competing situations.

Position	Group-Knockout			Home-Away			Non-Qualified-Qualified			Non-Qualified Opp.-Qualified Opp.			Draw/Lose-Win		
	Variable	Effect Size	Inference	Variable	Effect Size	Inference	Variable	Effect Size	Inference	Variable	Effect Size	Inference	Variable	Effect Size	Inference
CD				Clearance	0.22 ± 0.06	S *	Touch	0.47 ± 0.06	S ****	Touch	−0.39 ± 0.06	S ****	Touch	0.31 ± 0.07	S ****
				Pass	−0.22 ± 0.06	S *	Pass	0.50 ± 0.06	S ****	Pass	−0.39 ± 0.06	S ****	Pass	0.33 ± 0.07	S ****
							PA	0.29 ± 0.06	S ***	AW	−0.26 ± 0.06	S **			
							AccLB	0.21 ± 0.06	S *						
FB				Clearance	0.23 ± 0.07	S *	Touch	0.44 ± 0.07	S ****	Touch	−0.35 ± 0.07	S ****	Assist	0.39 ± 0.07	S ****
				Cross	−0.22 ± 0.07	S *	PA	0.32 ± 0.07	S ****	Pass	−0.38 ± 0.07	S ****	Touch	0.32 ± 0.07	S ****
							Pass	0.50 ± 0.07	S ****	AccLB	−0.22 ± 0.07	S *	Pass	0.39 ± 0.07	S ****
													PA	0.27 ± 0.07	S ***
WM	Foul	0.21 ± 0.11	S *	Shot	−0.26 ± 0.10	S **	Touch	0.59 ± 0.10	S ****	Touch	−0.42 ± 0.10	S ****	Assist	0.47 ± 0.12	S ****
				KP	−0.28 ± 0.10	S **	Pass	0.56 ± 0.10	S ****	Pass	−0.40 ± 0.10	S ****	Pass	0.51 ± 0.11	S ****
				Cross	−0.21 ± 0.10	S *	ThB	0.35 ± 0.10	S ***	Shot	−0.26 ± 0.10	S **	ShotOT	0.38 ± 0.11	S ****
				AccCross	−0.21 ± 0.10	S *	PA	0.30 ± 0.10	S ***	KP	−0.24 ± 0.10	S **	Touch	0.55 ± 0.11	S ****
							AccThB	0.26 ± 0.10	S **	ShotOT	−0.24 ± 0.10	S *	Shot	0.31 ± 0.11	S ***
							Shot	0.24 ± 0.10	S **	PA	−0.21 ± 0.10	S *	PA	0.31 ± 0.11	S ***
							ShotOT	0.29 ± 0.10	S **				KP	0.29 ± 0.11	S **
							Assist	0.21 ± 0.10	S *				ThB	0.27 ± 0.11	S **
							KP	0.21 ± 0.10	S *				AccThB	0.26 ± 0.11	S **
							AccLB	0.20 ± 0.10	S *				AccLB	0.23 ± 0.11	S *
CM				Shot	−0.20 ± 0.06	S *	Touch	0.49 ± 0.06	S ****	Touch	−0.39 ± 0.06	S ****	Assist	0.33 ± 0.07	S ****
							PA	0.35 ± 0.06	S ****	Pass	−0.37 ± 0.06	S ****	Touch	0.39 ± 0.06	S ****
							Pass	0.49 ± 0.06	S ****	LB	−0.23 ± 0.06	S ***	Pass	0.38 ± 0.06	S ****
							ThB	0.24 ± 0.06	S **				PA	0.21 ± 0.06	S *
							Assist	0.21 ± 0.06	S *						
FW				Shot	−0.32 ± 0.10	S ***	Touch	0.47 ± 0.10	S ****	Shot	−0.36 ± 0.10	S ****	Assist	0.46 ± 0.11	S ****
				KP	−0.28 ± 0.10	S **	Pass	0.43 ± 0.10	S ****	ShotOT	−0.32 ± 0.10	S ***	Shot	0.52 ± 0.10	S ****
				ShotOT	−0.22 ± 0.10	S *	Shot	0.32 ± 0.10	S ***	KP	−0.35 ± 0.10	S ***	ShotOT	0.57 ± 0.10	S ****
				AccCross	−0.23 ± 0.10	S *	ShotOT	0.32 ± 0.10	S ***	Assist	−0.28 ± 0.10	S **	Touch	0.46 ± 0.10	S ****
							Assist	0.28 ± 0.10	S **	Touch	−0.28 ± 0.10	S **	Pass	0.42 ± 0.10	S ****
							KP	0.30 ± 0.10	S **	Pass	−0.26 ± 0.10	S **	ThB	0.37 ± 0.11	S ****
							PA	0.28 ± 0.10	S **	ThB	−0.28 ± 0.10	S **	KP	0.34 ± 0.10	S ***
							Dribble	0.21 ± 0.10	S *	AccThB	−0.27 ± 0.10	S **	AccThB	0.33 ± 0.11	S ***
													Dribble	0.28 ± 0.10	S **

Note: Effect sizes are presented as the magnitude of the true difference in means ± 90% confidence interval, only the variables that showed clear differences were included. Positive effect size indicates that the mean values of variables from group A bigger than the mean values of variables from group B, negative effect size indicates that the mean values of variables from group B bigger than the mean values of variables from group A, e.g., group B-group A: group stage-knockout stage. Letters in parentheses denote the magnitude: t = trivial; s = small. Asterisks indicate the likelihood for the magnitude of the true difference in means as follows: * possible; ** likely; *** very likely; **** most likely. Abbreviations: AccCross = accurate cross pass; AccLB = accurate long ball; AccThB = accurate through; AW = aerial won; CD = central defender; CM = central midfielder; FB = full back; FW = forward; KP = key pass; PA = pass accuracy in %; ShotOT = shot on target; ThB = through ball; ball WM = wide midfielder.

## Supplementary Materials

The following are available online at https://www.mdpi.com/1660-4601/17/2/604/s1, Table S1. Descriptive statistics of match performance profiles of central defenders under five competing situations. Table S2. Descriptive statistics of match performance profiles of full backs under five competing situations. Table S3. Descriptive statistics of match performance profiles of wide midfielders under five competing situations. Table S4. Descriptive statistics of match performance profiles of central midfielders under five competing situations. Table S5. Descriptive statistics of match performance profiles of forwards under five competing situations.
