# Supplementary material for: Evaluation of the Technical Performance of Football Players in the UEFA Champions League
Source: Int J Environ Res Public Health. 2020 Jan 17;17(2):604. doi: 10.3390/ijerph17020604 (PMC7013673; doi:10.3390/ijerph17020604)
Supplement: Supplementary file 1 [file ijerph-17-00604-s001.pdf]

Supplementary Materials

Table S1. Descriptive statistics of match performance profiles of central defenders under five competing situations

Variable	Central Back									
	Group (n=2667)	Knockout (n=727)	Home (n=1328)	Away (n=1339)	Non-qualified (n=1365)	Qualified (n=1302)	Non-qualified Opp. (n=1300)	Qualified Opp. (n=1367)	Draw/Lose (n=1655)	Win (n=1012)
Assist	0.03±0.18	0.04±0.2	0.03±0.19	0.03±0.18	0.02±0.14	0.04±0.21	0.04±0.21	0.02±0.15	0.02±0.13	0.06±0.24
YC	0.19±0.39	0.21±0.41	0.17±0.37	0.21±0.41	0.2±0.4	0.18±0.38	0.18±0.38	0.2±0.4	0.21±0.41	0.16±0.36
Shot	0.58±0.9	0.53±0.93	0.67±0.95	0.49±0.83	0.53±0.86	0.63±0.93	0.63±0.91	0.53±0.89	0.54±0.89	0.64±0.91
ShotOT	0.16±0.42	0.16±0.46	0.2±0.46	0.13±0.37	0.15±0.41	0.17±0.43	0.17±0.43	0.15±0.41	0.14±0.4	0.2±0.46
Disp	0.41±0.82	0.4±0.75	0.38±0.78	0.44±0.86	0.39±0.81	0.43±0.83	0.41±0.81	0.41±0.83	0.42±0.85	0.39±0.78
UnsTouch	0.34±0.7	0.37±0.67	0.34±0.72	0.35±0.67	0.35±0.75	0.34±0.64	0.33±0.63	0.35±0.75	0.36±0.73	0.31±0.63
TT	2.02±1.7	2.23±1.84	2.03±1.73	2.01±1.68	1.96±1.64	2.07±1.77	2.03±1.77	2±1.64	2.03±1.65	2.01±1.79
Interception	2.4±1.79	2.26±1.75	2.4±1.8	2.41±1.78	2.42±1.83	2.39±1.75	2.38±1.82	2.42±1.77	2.43±1.78	2.36±1.81
Clearance	4.7±3.51	4.81±3.74	4.31±3.23	5.1±3.72	4.98±3.58	4.41±3.41	4.63±3.44	4.77±3.57	4.82±3.61	4.51±3.33
BS	0.62±0.87	0.61±0.84	0.54±0.81	0.7±0.92	0.67±0.93	0.56±0.8	0.58±0.83	0.65±0.91	0.64±0.9	0.58±0.82
Foul	1.09±1.12	1.05±1.09	1.09±1.13	1.1±1.11	1.09±1.13	1.1±1.11	1.06±1.11	1.12±1.13	1.14±1.15	1.02±1.07
Fouled	0.81±1.05	0.74±1.02	0.82±1.05	0.8±1.05	0.74±1.01	0.87±1.09	0.85±1.07	0.76±1.04	0.8±1.05	0.81±1.06
AW	1.52±1.65	1.66±1.69	1.58±1.71	1.46±1.59	1.39±1.55	1.65±1.74	1.74±1.78	1.31±1.49	1.46±1.65	1.62±1.66
Dribble	0.42±0.84	0.35±0.68	0.42±0.86	0.41±0.81	0.43±0.86	0.4±0.82	0.44±0.89	0.39±0.78	0.41±0.83	0.43±0.85
Offside	0.06±0.26	0.07±0.3	0.07±0.27	0.05±0.24	0.06±0.25	0.06±0.26	0.05±0.25	0.06±0.26	0.06±0.25	0.06±0.26
Touch	61.38±20.47	60.48±22.99	63.32±21.38	59.46±19.34	56.86±18.6	66.12±21.26	65.39±21.02	57.56±19.18	58.91±19.14	65.41±21.89
KP	0.41±0.83	0.36±0.77	0.46±0.85	0.36±0.81	0.39±0.8	0.43±0.87	0.43±0.86	0.39±0.81	0.39±0.81	0.45±0.88
PA	83.26±9.94	82.15±11.35	83.92±9.66	82.61±10.18	81.72±10.35	84.88±9.23	84.03±9.64	82.53±10.17	82.44±10.03	84.6±9.66
Pass	47.36±20.7	46.48±22.85	49.61±21.54	45.12±19.59	42.56±18.43	52.38±21.74	51.43±21.62	43.48±19	44.69±19.1	51.72±22.42
Cross	0.8±1.91	0.63±1.67	0.9±2.05	0.7±1.76	0.84±2.01	0.76±1.79	0.82±1.94	0.78±1.88	0.84±1.95	0.74±1.84
AccCross	0.17±0.57	0.14±0.51	0.2±0.63	0.14±0.49	0.17±0.59	0.17±0.55	0.19±0.61	0.16±0.53	0.17±0.56	0.18±0.59
LB	6.15±4.14	5.99±4.11	6.37±4.26	5.94±4.01	5.9±4.04	6.42±4.24	6.49±4.27	5.83±3.99	6.03±4.12	6.34±4.18
AccLB	3.69±3.11	3.43±3.11	3.94±3.24	3.45±2.96	3.39±2.97	4.01±3.23	3.96±3.22	3.44±2.98	3.53±3.05	3.96±3.2
ThB	0.11±0.43	0.13±0.5	0.11±0.46	0.1±0.39	0.08±0.32	0.14±0.51	0.13±0.46	0.09±0.39	0.09±0.38	0.14±0.49
AccThB	0.04±0.23	0.04±0.21	0.05±0.24	0.04±0.22	0.03±0.18	0.06±0.27	0.05±0.23	0.04±0.22	0.03±0.19	0.06±0.28

Note: Values are presented as mean ± SD. Abbreviations: ShotOT=shot on target; Disp=player is dispossessed on the ball by an opponent-no dribble involved; UnsTouch=Unsuccessful touch; AW=aerial won; YC=yellow card; TT=total tackle; BS=blocked shot; KP=key pass; PA=pass accuracy in %; AccCross=accurate cross pass; LB=long ball; AccLB=accurate long ball; ThB=through ball; AccThB=accurate through ball.

Table S2. Descriptive statistics of match performance profiles of full backs under five competing situations

Variable	Full Back									
	Group (n=2505)	Knockout (n=663)	Home (n=1273)	Away (n=1232)	Non-qualified (n=1279)	Qualified (n=1226)	Non-qualified Opp. (n=1250)	Qualified Opp. (n=1255)	Draw/Lose (n=1520)	Win (n=985)
Assist	0.07±0.27	0.08±0.27	0.08±0.29	0.06±0.24	0.05±0.21	0.1±0.31	0.09±0.31	0.05±0.22	0.03±0.16	0.14±0.37
YC	0.17±0.37	0.18±0.39	0.14±0.35	0.19±0.4	0.18±0.38	0.16±0.36	0.15±0.36	0.18±0.38	0.18±0.39	0.14±0.35
Shot	0.52±0.8	0.48±0.79	0.58±0.85	0.46±0.73	0.52±0.81	0.51±0.79	0.53±0.81	0.5±0.78	0.48±0.77	0.57±0.84
ShotOT	0.15±0.4	0.11±0.35	0.16±0.41	0.13±0.38	0.14±0.38	0.15±0.41	0.16±0.42	0.13±0.38	0.11±0.34	0.2±0.47
Disp	0.59±0.85	0.63±0.9	0.56±0.85	0.62±0.86	0.57±0.82	0.61±0.88	0.59±0.89	0.59±0.81	0.6±0.85	0.58±0.86
UnsTouch	0.6±0.87	0.65±0.85	0.6±0.89	0.6±0.85	0.61±0.84	0.59±0.9	0.57±0.84	0.63±0.89	0.61±0.87	0.58±0.87
TT	2.46±1.81	2.53±1.81	2.42±1.79	2.5±1.82	2.44±1.81	2.48±1.81	2.48±1.84	2.44±1.77	2.49±1.8	2.41±1.81
Interception	2.21±1.7	2.27±1.75	2.15±1.66	2.26±1.74	2.19±1.71	2.22±1.69	2.18±1.69	2.23±1.71	2.24±1.69	2.16±1.72
Clearance	3.52±2.83	3.37±2.7	3.22±2.65	3.84±2.97	3.72±2.88	3.32±2.76	3.49±2.92	3.55±2.73	3.63±2.76	3.36±2.93
BS	0.36±0.65	0.39±0.7	0.32±0.6	0.4±0.7	0.4±0.69	0.31±0.61	0.32±0.62	0.4±0.68	0.4±0.68	0.3±0.6
Foul	1.18±1.16	1.11±1.16	1.15±1.1	1.21±1.22	1.25±1.21	1.11±1.1	1.17±1.13	1.2±1.19	1.22±1.2	1.11±1.09
Fouled	0.94±1.07	1.01±1.08	0.96±1.12	0.91±1.02	0.91±1.03	0.96±1.11	0.99±1.1	0.88±1.04	0.9±1.03	0.99±1.13
AW	1.08±1.33	1.13±1.3	1.08±1.28	1.07±1.37	1.02±1.3	1.13±1.35	1.17±1.37	0.98±1.28	1.03±1.28	1.15±1.39
Dribble	0.63±0.99	0.79±1.11	0.65±0.98	0.61±1	0.64±1.04	0.63±0.94	0.65±1.03	0.62±0.95	0.63±0.98	0.64±1
Offside	0.07±0.29	0.08±0.32	0.08±0.31	0.07±0.25	0.07±0.28	0.08±0.29	0.08±0.29	0.07±0.28	0.07±0.28	0.08±0.29
Touch	61.08±18.62	63.09±21	62.48±18.32	59.64±18.81	57.1±16.78	65.24±19.51	64.24±19.14	57.94±17.53	58.7±17.58	64.77±19.55
KP	0.72±0.99	0.75±1.08	0.82±1.05	0.63±0.92	0.64±0.93	0.8±1.05	0.8±1.06	0.64±0.91	0.65±0.94	0.83±1.06
PA	80.38±10.08	80.24±10.79	81.13±9.88	79.61±10.23	78.67±10.22	82.17±9.62	81.38±9.82	79.39±10.24	79.13±9.91	82.33±10.03
Pass	44.07±16.96	45.93±19.59	45.56±16.6	42.53±17.2	40.01±14.74	48.3±18.07	47.2±17.71	40.95±15.58	41.43±15.37	48.14±18.44
Cross	2.56±2.76	2.37±2.49	2.85±2.94	2.25±2.52	2.43±2.74	2.69±2.77	2.65±2.8	2.46±2.72	2.55±2.82	2.56±2.66
AccCross	0.56±0.95	0.51±0.83	0.65±1.03	0.47±0.86	0.5±0.9	0.62±1	0.62±1.03	0.5±0.87	0.52±0.91	0.62±1.01
LB	4.53±3.18	4.26±2.93	4.67±3.16	4.38±3.19	4.47±3.18	4.59±3.17	4.78±3.31	4.28±3.02	4.49±3.23	4.58±3.09
AccLB	2.47±2.35	2.24±2.11	2.66±2.4	2.28±2.27	2.3±2.23	2.65±2.45	2.72±2.48	2.22±2.18	2.34±2.31	2.67±2.39
ThB	0.07±0.28	0.09±0.32	0.08±0.3	0.05±0.25	0.05±0.24	0.08±0.31	0.07±0.27	0.06±0.28	0.05±0.25	0.08±0.31
AccThB	0.02±0.14	0.02±0.17	0.03±0.17	0.01±0.11	0.02±0.12	0.03±0.16	0.03±0.17	0.01±0.11	0.01±0.11	0.03±0.18

Note: Values are presented as mean ± SD. Abbreviations: ShotOT=shot on target; Disp=player is dispossessed on the ball by an opponent-no dribble involved; UnsTouch=Unsuccessful touch; AW=aerial won; YC=yellow card; TT=total tackle; BS=blocked shot; KP=key pass; PA=pass accuracy in %; AccCross=accurate cross pass; LB=long ball; AccLB=accurate long ball; ThB=through ball; AccThB=accurate through ball.

Table S3. Descriptive statistics of match performance profiles of wide midfielders under five competing situations

Variable	Wide Midfielder									
	Group (n=1053)	Knockout (n=291)	Home (n=519)	Away (n=534)	Non-qualified (n=578)	Qualified (n=475)	Non-qualified Opp. (n=504)	Qualified Opp. (n=549)	Draw/Lose (n=700)	Win (n=353)
Assist	0.12±0.36	0.13±0.36	0.12±0.37	0.12±0.35	0.08±0.3	0.16±0.42	0.14±0.39	0.1±0.33	0.06±0.25	0.24±0.5
YC	0.16±0.36	0.22±0.42	0.13±0.34	0.18±0.39	0.17±0.37	0.15±0.35	0.16±0.37	0.15±0.36	0.17±0.38	0.13±0.33
Shot	1.84±1.7	2.05±2.03	2.04±1.67	1.64±1.7	1.64±1.5	2.07±1.88	2.07±1.86	1.62±1.5	1.66±1.57	2.18±1.88
ShotOT	0.69±0.98	0.8±1.11	0.73±0.95	0.65±1	0.55±0.78	0.86±1.15	0.82±1.11	0.58±0.82	0.56±0.82	0.95±1.19
Disp	1.66±1.51	1.77±1.61	1.68±1.54	1.65±1.48	1.62±1.49	1.71±1.54	1.65±1.57	1.67±1.45	1.7±1.53	1.59±1.46
UnsTouch	1.34±1.42	1.29±1.42	1.35±1.44	1.33±1.4	1.36±1.44	1.33±1.39	1.34±1.43	1.35±1.41	1.42±1.49	1.19±1.26
TT	1.78±1.65	1.88±1.74	1.77±1.6	1.79±1.7	1.74±1.68	1.82±1.61	1.83±1.63	1.73±1.66	1.72±1.65	1.89±1.64
Interception	1.47±1.55	1.59±1.69	1.38±1.52	1.55±1.58	1.47±1.49	1.47±1.62	1.5±1.61	1.44±1.49	1.44±1.5	1.53±1.65
Clearance	1.08±1.62	1.18±1.73	0.99±1.63	1.15±1.6	1.03±1.55	1.13±1.7	1.11±1.66	1.04±1.57	1.02±1.47	1.18±1.87
BS	0.15±0.42	0.19±0.51	0.13±0.39	0.17±0.45	0.13±0.39	0.17±0.45	0.15±0.44	0.15±0.41	0.14±0.39	0.18±0.48
Foul	1.31±1.26	1.65±1.42	1.29±1.22	1.33±1.3	1.36±1.28	1.25±1.24	1.34±1.28	1.29±1.25	1.34±1.29	1.24±1.2
Fouled	1.57±1.43	1.6±1.53	1.62±1.46	1.52±1.4	1.55±1.44	1.58±1.43	1.67±1.46	1.47±1.4	1.57±1.44	1.56±1.42
AW	0.8±1.1	1.03±1.55	0.78±1.03	0.82±1.17	0.74±1.05	0.88±1.16	0.87±1.16	0.74±1.04	0.77±1.08	0.86±1.14
Dribble	1.26±1.65	1.53±1.79	1.32±1.74	1.21±1.56	1.12±1.4	1.44±1.9	1.4±1.8	1.14±1.49	1.18±1.62	1.43±1.71
Offside	0.37±0.79	0.41±0.86	0.4±0.83	0.34±0.75	0.37±0.8	0.38±0.79	0.38±0.75	0.36±0.83	0.36±0.79	0.41±0.8
Touch	57.39±21	59.74±21.62	59.41±22.29	55.42±19.48	52.1±18.21	63.82±22.34	62.27±22.98	52.9±17.87	53.46±17.99	65.17±24.15
KP	1.28±1.4	1.44±1.42	1.48±1.56	1.08±1.2	1.15±1.34	1.44±1.46	1.47±1.55	1.11±1.22	1.13±1.27	1.57±1.59
PA	78.72±10.69	78.16±10.85	79.13±10.52	78.33±10.84	77.13±11.08	80.66±9.85	79.99±10.1	77.56±11.08	77.47±10.71	81.22±10.21
Pass	40.43±19.48	41.23±19.4	41.88±20.74	39.03±18.08	34.74±16.52	46.14±21.23	45.62±21.72	36.6±16.27	36.98±16.24	47.28±23.23
Cross	2.62±2.99	2.66±3.14	2.95±3.24	2.3±2.68	2.43±2.84	2.85±3.15	2.71±3.09	2.53±2.89	2.47±2.86	2.92±3.22
AccCross	0.58±1.03	0.55±1.01	0.69±1.15	0.47±0.89	0.54±1.01	0.63±1.06	0.65±1.11	0.52±0.95	0.51±0.94	0.73±1.18
LB	3.25±3.4	3.25±3.86	3.45±3.65	3.05±3.13	3.06±3.37	3.48±3.43	3.51±3.65	3.01±3.14	3.05±3.24	3.64±3.69
AccLB	2.18±2.78	2.21±3.11	2.35±3.04	2.01±2.51	1.94±2.63	2.46±2.94	2.42±3.03	1.96±2.52	1.96±2.55	2.61±3.15
ThB	0.28±0.61	0.37±0.69	0.31±0.68	0.25±0.54	0.18±0.45	0.4±0.75	0.33±0.69	0.23±0.52	0.22±0.53	0.4±0.73
AccThB	0.11±0.35	0.14±0.35	0.13±0.41	0.09±0.29	0.07±0.29	0.16±0.42	0.14±0.4	0.08±0.3	0.08±0.29	0.18±0.45

Note: Values are presented as mean ± SD. Abbreviations: ShotOT=shot on target; Disp=player is dispossessed on the ball by an opponent-no dribble involved; UnsTouch=Unsuccessful touch; AW=aerial won; YC=yellow card; TT=total tackle; BS=blocked shot; KP=key pass; PA=pass accuracy in %; AccCross=accurate cross pass; LB=long ball; AccLB=accurate long ball; ThB=through ball; AccThB=accurate through ball.

Table S4. Descriptive statistics of match performance profiles of central midfielders under five competing situations

Variable	Central Midfielder									
	Group (n=2794)	Knockout (n=774)	Home (n=1398)	Away (n=1396)	Non-qualified (n=1376)	Qualified (n=1418)	Non-qualified Opp. (n=1398)	Qualified Opp. (n=1396)	Draw/Lose (n=1678)	Win (n=1116)
Assist	0.09±0.3	0.12±0.35	0.09±0.31	0.08±0.3	0.05±0.23	0.12±0.36	0.12±0.35	0.06±0.24	0.04±0.21	0.15±0.4
YC	0.18±0.39	0.23±0.42	0.15±0.36	0.22±0.41	0.2±0.4	0.17±0.37	0.16±0.37	0.21±0.41	0.21±0.41	0.15±0.35
Shot	1.2±1.44	1.19±1.54	1.35±1.55	1.05±1.3	1.13±1.31	1.27±1.55	1.31±1.53	1.09±1.32	1.15±1.33	1.28±1.58
ShotOT	0.38±0.72	0.41±0.71	0.43±0.78	0.32±0.65	0.34±0.64	0.42±0.79	0.43±0.77	0.33±0.65	0.33±0.65	0.45±0.8
Disp	1.08±1.25	1.11±1.39	1.04±1.22	1.12±1.28	1.09±1.26	1.07±1.24	1.06±1.25	1.1±1.25	1.14±1.28	0.99±1.2
UnsTouch	0.8±1.07	0.85±1.1	0.79±1.03	0.81±1.12	0.83±1.09	0.76±1.06	0.79±1.06	0.8±1.09	0.83±1.11	0.75±1.02
TT	2.37±1.89	2.44±1.93	2.4±1.92	2.34±1.85	2.29±1.85	2.45±1.93	2.36±1.92	2.38±1.86	2.27±1.88	2.52±1.9
Interception	2.04±1.78	2±1.72	2.09±1.81	1.98±1.74	2.01±1.81	2.06±1.74	2.04±1.77	2.03±1.79	1.99±1.78	2.1±1.77
Clearance	2.04±2.79	1.94±2.57	1.86±2.63	2.22±2.93	2.19±2.89	1.89±2.67	2.04±2.88	2.04±2.69	2.07±2.77	2±2.81
BS	0.34±0.66	0.35±0.69	0.28±0.58	0.39±0.71	0.36±0.67	0.31±0.64	0.32±0.65	0.35±0.66	0.35±0.66	0.32±0.65
Foul	1.43±1.3	1.59±1.46	1.39±1.28	1.46±1.32	1.43±1.29	1.42±1.31	1.41±1.26	1.45±1.33	1.43±1.3	1.43±1.29
Fouled	1.29±1.31	1.41±1.3	1.34±1.38	1.24±1.24	1.23±1.33	1.34±1.28	1.28±1.3	1.29±1.32	1.27±1.33	1.32±1.28
AW	0.96±1.3	1.09±1.45	0.97±1.28	0.95±1.32	0.94±1.31	0.98±1.29	1.07±1.39	0.85±1.19	0.91±1.27	1.04±1.33
Dribble	0.78±1.16	0.97±1.4	0.8±1.21	0.76±1.11	0.72±1.09	0.83±1.23	0.83±1.23	0.72±1.09	0.73±1.12	0.85±1.22
Offside	0.11±0.4	0.09±0.35	0.14±0.45	0.09±0.34	0.1±0.4	0.13±0.41	0.13±0.41	0.1±0.39	0.11±0.39	0.12±0.42
Touch	67.45±23.3	70.61±25.33	68.4±23.49	66.5±23.06	61.7±19.55	73.03±25.21	71.91±24.61	62.98±20.98	63.77±20.86	72.99±25.57
KP	1±1.28	1.09±1.33	1.09±1.37	0.9±1.17	0.88±1.18	1.11±1.36	1.1±1.33	0.89±1.22	0.91±1.18	1.12±1.4
PA	83.51±8.77	83.61±9.31	83.56±8.94	83.45±8.6	81.87±9.24	85.09±7.98	84.26±8.66	82.75±8.82	82.58±8.67	84.9±8.74
Pass	52.78±22.92	55.41±25.18	53.54±23.02	52.03±22.79	47.19±18.77	58.21±25.17	56.98±24.47	48.57±20.41	49.18±20	58.19±25.78
Cross	1.48±2.46	1.67±2.64	1.65±2.62	1.31±2.29	1.37±2.45	1.58±2.47	1.62±2.63	1.34±2.27	1.48±2.49	1.47±2.43
AccCross	0.37±0.85	0.43±0.88	0.4±0.89	0.33±0.82	0.32±0.77	0.41±0.92	0.42±0.95	0.31±0.74	0.35±0.81	0.39±0.92
LB	5.36±3.97	5.22±3.96	5.52±4.04	5.19±3.9	5.09±3.79	5.62±4.13	5.72±4.22	4.79±3.68	5.18±3.8	5.63±4.2
AccLB	3.75±3.27	3.71±3.3	3.93±3.3	3.58±3.23	3.43±2.96	4.06±3.52	4.05±3.58	3.45±2.9	3.54±3.04	4.07±3.57
ThB	0.28±0.65	0.37±0.79	0.3±0.71	0.27±0.6	0.21±0.53	0.36±0.75	0.32±0.67	0.25±0.63	0.25±0.63	0.33±0.68
AccThB	0.1±0.33	0.14±0.41	0.1±0.34	0.1±0.32	0.07±0.28	0.13±0.37	0.12±0.36	0.08±0.3	0.08±0.29	0.13±0.39

Note: Values are presented as mean ± SD. Abbreviations: ShotOT=shot on target; Disp=player is dispossessed on the ball by an opponent-no dribble involved; UnsTouch=Unsuccessful touch; AW=aerial won; YC=yellow card; TT=total tackle; BS=blocked shot; KP=key pass; PA=pass accuracy in %; AccCross=accurate cross pass; LB=long ball; AccLB=accurate long ball; ThB=through ball; AccThB=accurate through ball.

Table S5. Descriptive statistics of match performance profiles of forwards under five competing situations

Variable	Forward									
	Group (n=1103)	Knockout (n=331)	Home (n=553)	Away (n=550)	Non-qualified (n=497)	Qualified (n=606)	Non-qualified Opp. (n=570)	Qualified Opp. (n=533)	Draw/Lose (n=676)	Win (n=427)
Assist	0.18±0.45	0.18±0.44	0.22±0.51	0.15±0.38	0.11±0.34	0.23±0.52	0.24±0.53	0.12±0.33	0.1±0.3	0.31±0.6
YC	0.15±0.36	0.17±0.38	0.16±0.37	0.15±0.35	0.14±0.35	0.16±0.37	0.14±0.35	0.16±0.37	0.17±0.38	0.12±0.33
Shot	2.82±2.05	3.17±2.18	3.13±2.1	2.51±1.95	2.47±1.85	3.11±2.16	3.17±2.23	2.45±1.76	2.42±1.8	3.45±2.25
ShotOT	1.15±1.21	1.33±1.3	1.27±1.24	1.02±1.17	0.95±1.09	1.31±1.28	1.34±1.36	0.95±0.99	0.89±0.99	1.56±1.41
Disp	2.03±1.71	2.03±1.68	2.01±1.75	2.05±1.66	1.98±1.66	2.07±1.75	2.01±1.74	2.04±1.67	2.01±1.66	2.06±1.78
UnsTouch	1.82±1.71	1.74±1.52	1.86±1.69	1.78±1.73	1.87±1.65	1.78±1.76	1.81±1.8	1.83±1.61	1.88±1.75	1.72±1.65
TT	1.15±1.34	1.01±1.13	1.18±1.35	1.12±1.32	1.2±1.36	1.11±1.32	1.14±1.31	1.17±1.36	1.13±1.37	1.19±1.29
Interception	0.8±1.18	0.78±1.08	0.75±1.09	0.85±1.26	0.86±1.18	0.75±1.17	0.79±1.15	0.81±1.21	0.8±1.18	0.8±1.17
Clearance	0.55±0.89	0.6±1.01	0.51±0.87	0.59±0.92	0.59±0.93	0.51±0.86	0.54±0.92	0.56±0.86	0.54±0.89	0.56±0.9
BS	0.08±0.29	0.1±0.33	0.07±0.27	0.09±0.31	0.09±0.29	0.08±0.29	0.07±0.27	0.1±0.31	0.08±0.29	0.08±0.3
Foul	1.4±1.45	1.41±1.37	1.41±1.39	1.39±1.5	1.48±1.48	1.33±1.41	1.39±1.4	1.41±1.49	1.42±1.48	1.36±1.39
Fouled	1.74±1.54	1.95±1.6	1.67±1.48	1.81±1.6	1.67±1.54	1.8±1.54	1.87±1.61	1.6±1.44	1.71±1.52	1.78±1.57
AW	1.27±1.86	1.1±1.49	1.35±1.94	1.19±1.77	1.41±2.13	1.15±1.6	1.18±1.8	1.36±1.92	1.3±1.94	1.22±1.73
Dribble	1.36±1.7	1.98±2.35	1.38±1.8	1.34±1.61	1.17±1.5	1.51±1.84	1.52±1.9	1.19±1.44	1.16±1.41	1.68±2.05
Offside	0.73±1.04	0.66±1.04	0.81±1.07	0.65±0.99	0.71±1.14	0.75±0.94	0.72±1.05	0.74±1.02	0.7±1.08	0.79±0.97
Touch	51.65±20.35	55.68±22.64	52.14±20.7	51.16±20	46.96±16.48	55.5±22.34	54.29±21.52	48.83±18.63	48±17.65	56.43±22.88
KP	1.55±1.47	1.53±1.57	1.75±1.53	1.35±1.37	1.33±1.38	1.74±1.51	1.79±1.57	1.3±1.31	1.36±1.33	1.86±1.63
PA	77.31±11.1	78.68±11.36	76.96±11.13	77.66±11.07	75.41±11.17	78.86±10.8	77.78±10.87	76.8±11.32	76.88±10.82	77.98±11.5
Pass	34.95±16.82	37.97±19.2	35±16.77	34.91±16.89	31.07±12.94	38.14±18.86	36.94±17.72	32.83±15.55	32.21±14.44	39.09±19.25
Cross	2.28±2.96	2.02±2.58	2.52±3.29	2.04±2.57	2.17±3.04	2.36±2.9	2.4±2.95	2.14±2.98	2.19±2.96	2.41±2.97
AccCross	0.5±0.96	0.42±0.83	0.6±1.08	0.39±0.82	0.47±0.95	0.51±0.98	0.56±1.05	0.42±0.86	0.43±0.86	0.6±1.11
LB	1.85±2.1	1.69±1.92	1.77±2.07	1.92±2.14	1.76±2.11	1.92±2.1	1.99±2.23	1.7±1.94	1.74±2.08	2.02±2.13
AccLB	1.28±1.66	1.18±1.5	1.27±1.69	1.29±1.63	1.2±1.67	1.35±1.66	1.38±1.76	1.18±1.55	1.21±1.62	1.4±1.72
ThB	0.53±1.01	0.76±1.24	0.52±1	0.55±1.03	0.33±0.68	0.7±1.2	0.66±1.11	0.4±0.88	0.39±0.77	0.77±1.28
AccThB	0.21±0.55	0.3±0.65	0.22±0.58	0.21±0.5	0.12±0.37	0.29±0.64	0.28±0.64	0.14±0.41	0.14±0.4	0.33±0.71

Note: Values are presented as mean ± SD. Abbreviations: ShotOT=shot on target; Disp=player is dispossessed on the ball by an opponent-no dribble involved; UnsTouch=Unsuccessful touch; AW=aerial won; YC=yellow card; TT=total tackle; BS=blocked shot; KP=key pass; PA=pass accuracy in %; AccCross=accurate cross pass; LB=long ball; AccLB=accurate long ball; ThB=through ball; AccThB=accurate through ball.
